# DNN-Based Noise Reduction Significantly Improves Bimodal Benefit in Background Noise for Cochlear Implant Users

**DOI:** 10.3390/jcm14155302

**Published:** 2025-07-27

**Authors:** Courtney Kolberg, Sarah O. Holbert, Jamie M. Bogle, Aniket A. Saoji

**Affiliations:** 1 Department of Otolaryngology-Head and Neck Surgery, Division of Audiology, Mayo Clinic, Scottsdale, AZ 85259, USA; kolberg.courtney@mayo.edu (C.K.); holbert.sarah@mayo.edu (S.O.H.); bogle.jamie@mayo.edu (J.M.B.); 2 Department of Otolaryngology-Head and Neck Surgery, Division of Audiology, Mayo Clinic, Rochester, MN 55905, USA

**Keywords:** cochlear implant, bimodal, deep neural network, artificial intelligence, hearing aid

## Abstract

**Background/Objectives**: Traditional hearing aid noise reduction algorithms offer no additional benefit in noisy situations for bimodal cochlear implant (CI) users with a CI in one ear and a hearing aid (HA) in the other. Recent breakthroughs in deep neural network (DNN)-based noise reduction have improved speech understanding for hearing aid users in noisy environments. These advancements could also boost speech perception in noise for bimodal CI users. This study investigated the effectiveness of DNN-based noise reduction in the HAs used by bimodal CI patients. **Methods**: Eleven bimodal CI patients, aged 71–89 years old, were fit with a Phonak Audéo Sphere Infinio 90 HA in their non-implanted ear and were provided with a Calm Situation program and Spheric Speech in Loud Noise program that uses DNN-based noise reduction. Sentence recognition scores were measured using AzBio sentences in quiet and in noise with the CI alone, hearing aid alone, and bimodally with both the Calm Situation and DNN HA programs. **Results**: The DNN program in the hearing aid significantly improved bimodal performance in noise, with sentence recognition scores reaching 79% compared to 60% with Calm Situation (a 19% average benefit, *p* < 0.001). When compared to the CI-alone condition in multi-talker babble, the DNN HA program offered a 40% bimodal benefit, significantly higher than the 21% score seen with the Calm Situation program. **Conclusions**: DNN-based noise reduction in HA significantly improves speech understanding in noise for bimodal CI users. Utilization of this technology is a promising option to address patients’ common complaint of speech understanding in noise.

## 1. Introduction

Cochlear implants improve speech recognition for those with significant hearing loss who do not receive benefit from traditional acoustic amplification. Cochlear implants provide direct electrical stimulation to the auditory nerve via spiral ganglion cells, circumventing the damaged sensory hair cells within the cochlea that accompany advanced sensorineural hearing loss. Although cochlear implants provide a substantial benefit for speech understanding for those who meet candidacy criteria, cochlear implant recipients continue to report substantial difficulty understanding speech in background noise. Multiple studies have investigated the utility of cochlear implant programming techniques to improve speech intelligibility in background noise. Cochlear implant programming methods such as increased maxima for perimodiolar electrode arrays using N of M processing strategies [1], choice of paired or sequential stimulation mode [2], as well as individualized frequency allocation tables [3,4] may lead to improved speech understanding in noise. However, even with optimized cochlear implant programming, monaural cochlear implant stimulation provides relatively limited benefit compared to binaural stimulation.

Bimodal stimulation, which refers to the use of a cochlear implant in one ear and a hearing aid in the non-implanted ear, offers a practical solution for improving speech understanding in noisy environments for cochlear implant users. This stimulation approach is highly applicable for a large percentage of cochlear implant recipients, as roughly 85% have audiometric profiles suitable for bimodal stimulation [5]. Cochlear implant recipients with aidable acoustic hearing in the contralateral ear gain improved speech understanding in quiet and noise when fit with a hearing aid [6,7,8,9,10]. Zhang et al. [9] demonstrated that even a very limited acoustic bandwidth of just 250 Hz in the non-implanted ear can still provide a noticeable improvement for cochlear implant users. This finding suggests that even minimal residual hearing in the contralateral ear can contribute significantly to better sound perception. With an appropriately fit contralateral hearing aid, bimodal users can achieve up to 10–30% improvement on speech recognition tasks in both quiet and noisy listening conditions [6,7,11,12,13,14,15,16]. Therefore, the fitting characteristics and digital signal processing of the contralateral hearing aid are important clinical considerations when working with these patients.

To mitigate the effects of hearing loss, hearing aid fittings are routinely verified through real-ear measurements. This procedure ensures that the hearing aid’s output precisely aligns with the patient’s prescribed target gain. Despite these precise fittings, hearing aid users report persistent difficulties with speech comprehension, particularly in the presence of background noise. Advancement in hearing aid technologies has enabled the use of directional microphones and noise reduction algorithms to enhance the signal-to-noise ratio and improve speech comprehension. Notably, directional microphones are most effective in boosting the signal-to-noise ratio when the speech source and competing noise are spatially separated [17]. When hearing aids are well-fitted and the ear canal is occluded, directional microphones have been shown to provide a signal-to-noise ratio benefit ranging from 3 to 7 dB [18,19,20]. In the context of bimodal cochlear implant users, incorporating directional microphones on the hearing aid (without altering the cochlear implant settings) has led to notable improvements in speech perception [21,22]. For example, Michels et al. [21] demonstrated a 3.8 dB improvement in speech reception thresholds. Similarly, Stronks et al. [22] found a 2.6 dB improvement in speech reception thresholds when directional microphones were utilized in the hearing aid for bimodal cochlear implant users. While directional microphones offer clear benefits for speech perception in hearing aids, traditional noise reduction algorithms have shown more limited improvements. These algorithms are most effective with steady-state noises, which the noise reduction algorithms mitigate by predicting and attenuating the noise envelope. However, the effectiveness of these traditional noise algorithms significantly diminishes when faced with less predictable, fluctuating noises, such as multi-talker babble [23,24]. Similarly, in bimodal cochlear implant users, traditional noise reduction in the hearing aid has demonstrated no significant benefit for speech perception in background noise [17,23]. Directional microphones and noise reduction algorithms each offer unique advantages and disadvantages in improving speech understanding in noise. Directional microphones excel at enhancing the signal-to-noise ratio when speech and noise are spatially separated, providing benefits even in challenging zero or negative signal-to-noise ratio environments. However, their effectiveness is limited when speech and background noise are co-located. Conversely, traditional noise reduction algorithms can improve the signal to noise ratio for co-located steady-state noise, but they generally perform poorly in zero or negative signal to noise ratio conditions. Furthermore, they have shown limited benefits when faced with fluctuating background noise.

Deep Neural Network (DNN)-based noise reduction, an artificial intelligence driven form of noise reduction, has been shown to improve speech understanding in complex listening situations, such as fluctuating noise, multi-talker babble, and co-located noise [25,26]. The present study aims to assess the benefit of DNN-based noise reduction in bimodal cochlear implant users who make use of this technology in their non-implanted ear. We hypothesize that bimodal cochlear implant patients will demonstrate improved speech recognition in noise with DNN-based noise processing compared to traditional hearing aid noise reduction. To test the impact of DNN-based noise reduction in bimodal cochlear implant users, the contralateral ear of the bimodal cochlear implant patient was fit with a Phonak Audéo Sphere Infinio 90 receiver-in-canal hearing aid. This hearing aid employs a DNN-based noise reduction with a large network with 4.5 million parameters and is trained using a dataset of 22 million sound samples that covers a large range of noisy listening conditions. The Deep Neural Network processes the input sound signal by dividing it into 64 frequency bands and analyzing both the amplitude and phase components in each band. This comprehensive method allows the DNN to more accurately separate speech from noise than conventional noise reduction algorithms, which typically operate at lower frequency resolutions and utilize real-valued filter weights. By employing a complex-valued filter, the DNN can more precisely reconstruct the speech signal. This approach differs significantly from traditional methods that primarily reduce gain during pauses between words or in quieter segments, which can sometimes degrade audio quality and introduce distortion when reconstructing the audio signal. Because DNN evaluates the entire signal, it offers a more effective solution for reducing noise while preserving speech intelligibility [27].

Recently, Saoji et al. [26] showed that DNN noise reduction technology significantly improves speech understanding in multi-talker babble noise for potential cochlear implant candidates with moderate to profound sensorineural hearing loss. In that study, the speech and noise were co-located and presented through a loudspeaker located in front of the patient. The dramatic improvement in speech recognition demonstrated with DNN-based noise reduction calls into question current cochlear implant candidacy guidelines and has the potential to delay or avoid cochlear implantation for patients with significant amount of residual hearing. What remains unknown is how this technology can be further utilized in patients with advanced levels of hearing loss and how it will impact cochlear implant patients who have aidable residual hearing in the contralateral ear. In these bimodal patients, the contralateral hearing aid provides low-frequency information that complements the cochlear implant signal and has been shown to provide significant benefit over the cochlear implant alone. While the traditional noise reduction in the hearing aid has failed to show any speech perception benefit in noisy listening conditions the advances in the DNN-based noise reduction technology in hearing aids may provide a substantial benefit for speech understanding in noise for bimodal cochlear implant patients. However, this potential benefit remains unexplored. In the present study, we report on the impact of DNN-based noise reduction on bimodal cochlear implant patients.

## 2. Materials and Methods

**Figure 1:**
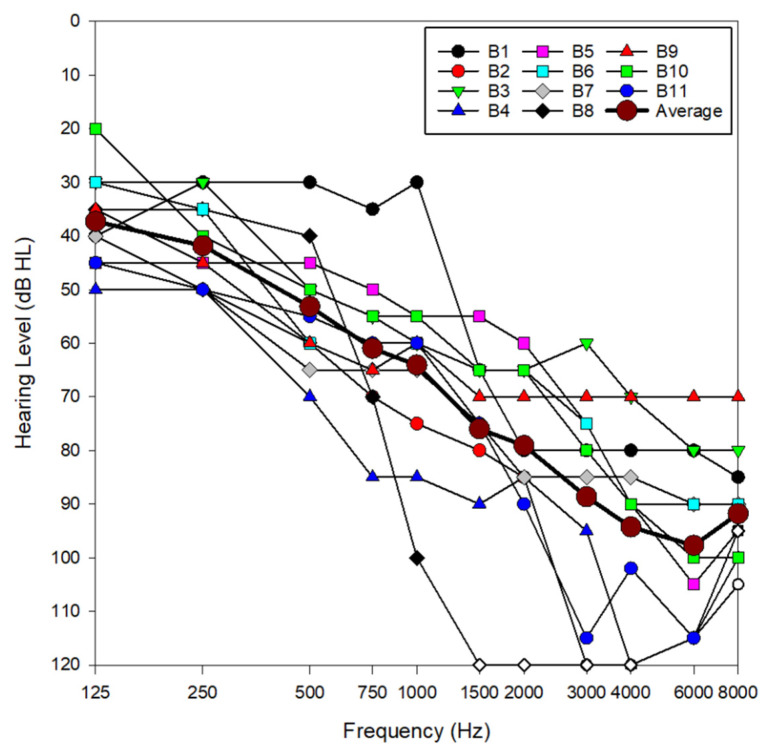
Audiometric data for all subjects in the non-implanted ear, as well as average audiometric thresholds across subjects. Open symbols indicate no response at the limits of the audiometer.

This study explored the effectiveness of DNN-based noise reduction in hearing aids used by 11 bimodal cochlear implant patients between the ages of 71 and 89 years old. All participants routinely used a cochlear implant in one ear and a hearing aid in the other. During a standard clinical visit, these patients were fitted with a Phonak Audéo Sphere Infinio 90 receiver-in-canal hearing aid (Stäfa, Switzerland), equipped with power domes. Each hearing aid was programmed with two manual programs: (1) Calm Situation program: This setting used real ear sound with microphone characteristics similar to those provided by the pinna and did not apply any noise reduction algorithms. (2) Spheric speech in loud noise program: This setting utilized DNN-based noise reduction to improve speech understanding in noisy environments. Patients had varying degrees of hearing loss in their non-implanted ear, with an average low-frequency pure tone average (LF-PTA; 125, 250, 500 and 1000 Hz) of 50 dB HL (Figure 1). In Figure 1, open symbols represent no responses from the patient at that presentation level. See Table 1 for detailed subject demographics. This study was approved by the Mayo Clinic Institutional Review Board (#25-001745). Speech perception scores reported in this study were obtained between 6 May 2025 and 1 June 2025.

Sentence recognition scores were evaluated both in a quiet environment and with background noise. For each test condition, a list of 20 AzBio sentences was presented at 60 dB SPL via a loudspeaker positioned at 0 degrees azimuth. To assess speech recognition in noise, multi-talker babble (referred to as MTB in noisy test conditions) was introduced. For patients B2 through B11, the multi-talker babble was presented at 55 dB SPL, resulting in a signal-to-noise ratio of 5 dB. For patient B1, a signal to noise ratio of 0 dB was used (multi-talker babble at 60 dB SPL) to prevent a ceiling effect, where performance is too high to show meaningful differences. In all cases, the multi-talker babble was presented from the same loudspeaker as the sentences, creating a co-located speech and noise test condition. When measuring speech perception in the presence of intermittent noise, a common practice is to start the noise only a few milliseconds before a sentence begins and end it shortly after the sentence concludes. This brief overlap does not provide sufficient time for noise reduction algorithms to effectively optimize the signal-to-noise ratio. During the tests reported in this study, multi-talker babble noise was played continuously throughout the presentation of an entire list of 20 AzBio sentences.

Sentence recognition scores were measured using the following listening conditions (1) CI: In this condition, only the patient’s cochlear implant was active, using their daily listening program. The opposite ear was plugged. (2) HA_Calm_: Here, the hearing aid alone was active, set to the “Calm Situation” program. The cochlear implant processor was removed from the implanted ear, and a plug was inserted to ensure only the hearing aid was contributing to sound perception. (3) HA_DNN_: As with the other hearing aid condition, this condition involved the hearing aid acting alone in the non-implanted ear and the cochlear implant sound processor removed. The hearing aid was set to the DNN-based noise reduction program. (4) Bimodal_Calm_: This bimodal condition involved the patient’s cochlear implant operating with their personal program in the implanted ear, while the hearing aid in the opposite ear was set to the “Calm Situation” program (5) Bimodal_DNN_: In this bimodal condition, the patient’s cochlear implant was active with their personal program, and the hearing aid in the opposite ear utilized the DNN-based noise reduction program. To assess the benefit of bimodal listening and DNN-based noise reduction in bimodal cochlear implant users, sentence recognition was measured in (1) CI-Quiet (2) CI-MTB (3) Bimodal_Calm_-Quiet (4) Bimodal_Calm_-MTB (5) Bimodal_DNN_-MTB. Statistical analyses were performed using SigmaPlot (version 14.5; San Jose, CA, USA). To analyze sentence recognition scores in the cochlear implant alone and bimodal conditions, statistical analysis was performed as follows. Data normality was assessed with the Shapiro–Wilk test. Kruskal–Wallis ANOVA on ranks was used to compare speech performance across conditions. Post hoc pairwise comparisons were conducted using the Student–Newman–Keuls test. To determine the benefit of DNN in the hearing aid alone conditions (without the cochlear implant), we also measured sentence recognition in the following scenarios (1) HA_Calm_-Quiet (2) HA_Calm_-MTB and (3) HA_DNN_-MTB. For statistical analysis, the Shapiro–Wilk test was used to test for normality. One way analysis was used to compare sentence recognition across the different test conditions and the Student–Newman–Keuls test was used for pairwise comparison. To prevent systematic order effects, test conditions were randomized when measuring sentence recognition scores. Additional statistical analyses were conducted using Spearman correlation to assess potential relationships between patient variables and speech recognition scores.

## 3. Results

**Figure 2:**
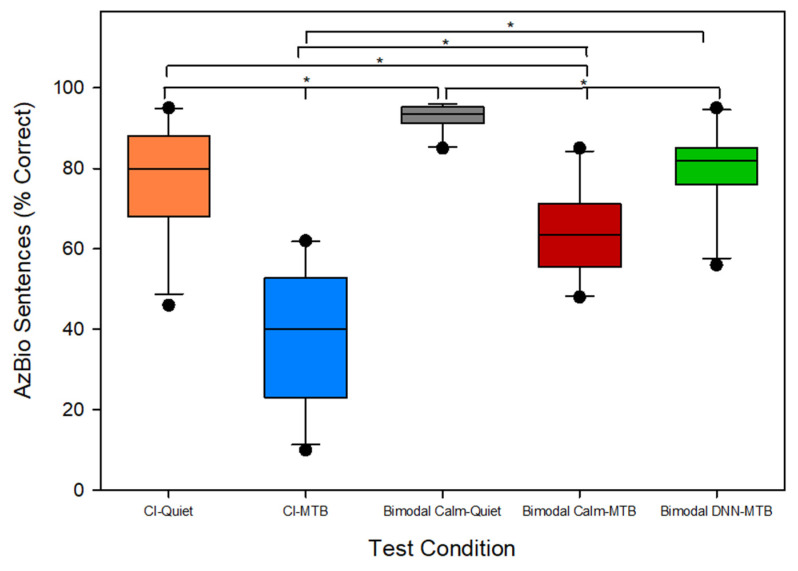
Average sentence recognition scores in quiet and in the presence of multi-talker babble for the 11 bimodal (CI + HA) patients across the five different test conditions (1) CI-Quiet (2) CI-MTB (3) Bimodal_Calm_-Quiet (4) Bimodal_Calm_-MTB (5) Bimodal_DNN_-MTB. CI: Cochlear implant; HA: Hearing aid; MTB: Multi-talker babble; DNN: Deep Neural Network. All pairwise comparisons were statistically significant (* *p* < 0.005) except the comparison between CI-Quiet and Bimodal_DNN_-MTB.

Figure 2 illustrates the average sentence recognition scores, measured using AzBio sentences, across five distinct listening conditions (CI-Quiet, CI-MTB, Bimodal_Calm_-Quiet, Bimodal_Calm_-MTB, and Bimodal_DNN_-MTB). The x-axis represents the different test conditions, while the y-axis displays the AzBio sentence recognition scores as the percentage of words correctly recognized. For the 11 cochlear implant patients, the average sentence recognition score in CI-Quiet was 78%. The introduction of multi-talker babble noise in the CI-MTB condition with a signal-to-noise ratio of 0 or 5 dB decreased sentence recognition to 39% on average. In the Bimodal_Calm_-Quiet bimodal condition, in which patients used a cochlear implant in one ear and a hearing aid set to the Calm Situation program in the other, the average sentence recognition in quiet increased to 93%. This represents a 15% (difference between 93 and 78%) improvement over the CI-Quiet condition, highlighting the benefit of bimodal hearing. However, when multi-talker babble noise was introduced, the bimodal score (Bimodal_Calm_-MTB) dropped to an average of 60%. Switching the hearing aid to the DNN noise reduction program within the bimodal setup (Bimodal_DNN_-MTB) led to a substantial improvement in sentence recognition, bringing the average score up to 79%. This represents a 19% increase in sentence recognition score from the use of DDN-based noise reduction in the hearing aid ear in bimodal cochlear implant patients. Kruskal–Wallis ANOVA showed significant differences [*H*(4) = 38.256, *p* < 0.001] across the five test conditions shown in Figure 2. The Student–Newman–Keuls test was used for pairwise comparisons with a Bonferroni correction (with α = 0.005 across 10 pairs). The results showed significant differences (*p* < 0.005) among all test conditions. The sole exception was the comparison between the CI-quiet and Bimodal_DNN_-MTB conditions, which yielded a *p*-value of 0.844. These findings show that adding a hearing aid to the contralateral ear significantly enhances speech perception for bimodal cochlear implant patients. In the presence of multi-talker babble, when the hearing aid was set to the Calm Situation program (Bimodal_Calm_-MTB), bimodal hearing provided a 21% improvement in sentence recognition compared to using the cochlear implant alone. Use of a hearing aid with a DNN-based noise reduction program in the contralateral ear provided a bimodal benefit of 40% in noisy listening conditions for cochlear implant patients. Thus, integrating a DNN-based noise reduction system, like the one in this study, into the hearing aid can further boost speech perception for cochlear implant patients in noisy conditions.

**Figure 3:**
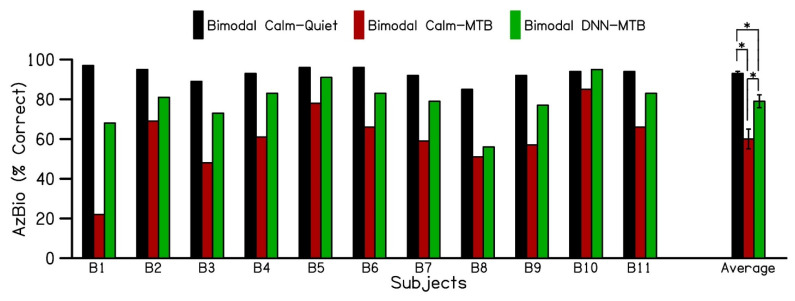
Individual and average performance of bimodal (CI + HA) users on AzBio sentences, evaluated in three different bimodal conditions. (1) CI + HA in calm situation program in quiet (Bimodal Calm–Quiet), (2) CI + HA in calm situation program in the presence of multi-talker babble (Bimodal Calm–MTB), (3) CI + HA in DNN noise reduction program in the presence of multi-talker babble (Bimodal DNN–MTB). CI: Cochlear implant; HA: Hearing aid; MTB: Multi-talker babble; DNN: Deep Neural Network. (* *p* < 0.005).

Figure 3 displays the individual and average AzBio speech recognition scores for 11 bimodal cochlear implant patients. These scores were measured in quiet and multi-talker babble environments across three test conditions: Bimodal_Calm_-Quiet, Bimodal_Calm_-MTB, and Bimodal_DNN_-MTB. The error bars indicate the standard error of the mean. Patients using both their cochlear implant and a hearing aid in the Calm Situation program (Bimodal_Calm_-Quiet) achieved an average sentence recognition score of 93% (black bars) in quiet. However, when multi-talker babble noise was introduced, the bimodal sentence recognition scores (Bimodal_Calm_ -MTB) decreased to 60% (red bars). Switching the hearing aid program to DNN noise reduction (Bimodal_DNN_-MTB) improved the average bimodal sentence recognition score to 79% (green bars) for the 11 cochlear implant patients in noisy environments. This shows that using DNN noise reduction in the hearing aid can enhance sentence recognition by an average of 19% for bimodal cochlear implant patients in noisy listening conditions compared to standard hearing aid technology. Furthermore, as depicted in Figure 3, all patients demonstrated some level of increased benefit when activating DNN-based noise reduction.

**Figure 4:**
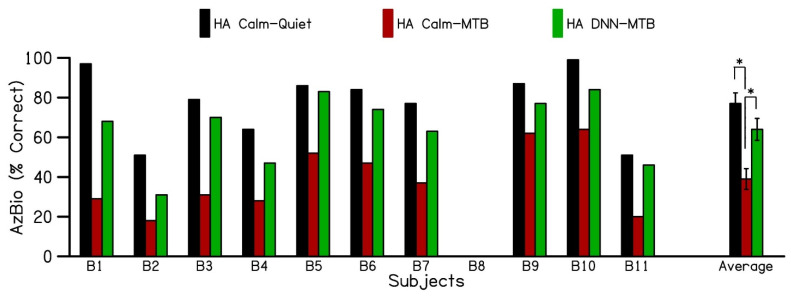
Individual and average performance of the hearing aid alone on AzBio sentences, evaluated in three different conditions. (1) HA in calm situation program in quiet (HA Calm–Quiet), (2) HA in calm situation program in the presence of multi-talker babble (HA Calm–MTB) (3), HA in DNN noise reduction program in the presence of multi-talker babble (HA DNN–MTB). HA: Hearing aid; DNN: Deep Neural Network; MTB: Multi-talker babble. (* *p* < 0.017).

Figure 4 shows the individual and average speech recognition scores for the hearing alone in the contralateral ear (without the cochlear implant) in quiet and in the presence of multi-talker babble noise. Sentence recognition scores were not available for subject B8 with the hearing aid alone. The results show an average sentence recognition score of 78% (black bars) for hearing alone in the Calm Situation program in the quiet condition (HA_Calm_-Quiet). The addition of multi-talker babble noise with the hearing aid alone in the Calm Situation program (HA_Calm_-MTB) decreased sentence recognition to 39%. Switching to the DNN noise reduction program in the hearing aid alone (HA_DNN_-MTB) condition increased sentence recognition scores to 64%, demonstrating a 25% increase in sentence recognition in the presence of multi-talker babble with the use of DNN-based noise reduction. The sentence recognition scores for the hearing aid alone conditions passed the Shapiro–Wilk test for normality (*p* = 0.262). A one-way analysis of variance revealed a statistically significant difference in sentence recognition scores across the different test conditions (F (2,27) = 13.231, *p* < 0.001). Post hoc pairwise comparisons, performed using the Student–Newman–Keuls test with a Bonferroni correction (α = 0.017 across 3 pairs), showed significant differences (*p* < 0.017) among all test conditions. The only exception was the comparison between the HA_Calm_-Quiet and HA_DNN_-MTB conditions. These findings indicate that using the hearing aid alone in the Calm Situation program leads to a significant decrease in sentence recognition when multi-talker babble is introduced, compared to quiet conditions. However, switching the hearing aid to the DNN program significantly improved speech perception in the presence of multi-talker babble, with scores approaching those achieved with the hearing aid alone in quiet.

Spearman correlation was used to determine any relationship between audiometric thresholds and improvement in sentence recognition with the use of DNN-based noise reduction in the hearing aid used in the contralateral ear. Unsurprisingly, no relationship was found between LF-PTA and DNN benefit in the bimodal condition (r = −0.172, *p =* 0.595). However, a negative relationship was identified between DNN benefit in the hearing aid alone condition and LF-PTA (r = −0.671, *p* = 0.03), indicating those patients with better low-frequency thresholds obtained greater benefit from the use of DNN-based noise reduction in their hearing aid ear when solely relying on that hearing aid.

## 4. Discussion

Patients with a bimodal hearing configuration obtain significant benefit for speech understanding in noise compared to performance with monaural CI stimulation [6,7,8,9,10]. However, traditional bimodal stimulation has limitations with improving speech intelligibly in environments with poor signal to noise ratios and/or co-located speech and noise. Our results suggest that DNN-based noise reduction further improves speech intelligibility in challenging co-located speech and multi-talker babble noise situations when applied to the hearing aid in the non-implanted ear.

For individuals with cochlear implants who also have aidable residual hearing in the opposite ear, using a contralateral hearing aid has been shown to improve sentence recognition by 10% to 30% in both quiet and noisy environments [6,7,11,12,13,14,15,16]. In our current study, we observed a bimodal benefit of 15% in quiet and 21% in the presence of multi-talker babble noise when compared to using the cochlear implant alone. When using the hearing aid with a DNN-based noise reduction program, the bimodal benefit increased from 21% to 40% in the presence of multi-talker babble, compared to using the cochlear implant alone. Our bimodal benefit results with a traditional hearing aid in noise align with previous research, however our findings with the DNN-based noise reduction substantially exceed the speech in noise benefit with bimodal stimulation previously reported.

While directional microphones in the hearing aid are known to enhance sentence recognition when speech and noise are spatially separated, traditional noise reduction algorithms in hearing aids have demonstrated limited improvements in sentence recognition scores [21,22]. Our current findings indicate that a DNN-based noise reduction algorithm offers superior performance compared to standard noise reduction algorithms used in hearing aids. This study was conducted using co-located speech and noise stimuli where the AzBio sentences and the multi-talker babble were presented through a single loudspeaker located in the front of the cochlear implant patient (i.e., 0 degrees azimuth). This test setup prohibits directional microphones from providing benefit [20] and relies solely on DNN-based noise reduction for signal to noise ratio improvement. Additional speech recognition benefits may be observed in spatially separated testing configurations that utilize DNN-based noise reduction plus directional microphones. Raufer et al. [25] corroborates this, as they showed a 5.5 dB signal to noise ratio improvement with DDN-based noise processing alone that increased to 8.5 dB signal to noise ratio when directional microphones were active. Further work is needed to fully understand the DNN bimodal benefit in this more realistic, multi-speaker testing configuration.

Traditional bimodal benefit in complex noise has been attributed to binaural summation and glimpsing, in which the acoustic ear is able to hear the target signal through temporal dips in the background noise [28,29,30]. However, as DNN-based noise reduction better identifies and reduces competing background noise, the target signal is considerably more audible to the acoustic ear, improving the patient’s ability to “glimpse” the signal. This may account for the vast improvement in speech recognition in noise observed with DNN-based noise reduction in bimodal users, especially as the hearing aid ear appears to drive the benefit. Throughout testing bimodal conditions in our study, the cochlear implant program was unchanged, whereas the hearing aid settings varied. Even with DNN-based noise reduction applied only to the non-implanted ear, significant speech recognition benefit was observed. These findings contradict the conventional belief that the cochlear implant is the primary driver for speech clarity, whereas the hearing aid’s role is to support sound quality [31], particularly in noisy environments. Thus, these results suggest that fitting a hearing aid utilizing DNN-based noise reduction in a patient’s non-implanted ear may be a useful method to address the common challenge of speech understanding in noise.

Our results with the hearing aid alone further confirm the key role that the acoustic ear plays with speech understanding in noise. Patient performance in noise with the DNN hearing aid program was equivalent to performance in quiet (Figure 3). On average, patients realized a 25% improvement in speech in noise understanding when activating DNN-based noise reduction compared to standard hearing aid noise reduction technology, which Saoji et al. [26] also describes. Unsurprisingly, the amount of acoustic hearing in the non-implanted ear was not associated with DNN benefit bimodally, as bimodal benefit can be observed with a limited acoustic bandwidth [9]. Interestingly, these patients also had very poor unaided word recognition scores yet obtained significant benefit with DNN-based noise reduction in their presumably poorer, degraded acoustic ear, even with the hearing aid alone. Like Saoji et al. our results suggest DNN-based noise reduction provides improved speech clarity in noise even for patients who may not traditionally benefit from a hearing aid.

Further work is needed to fully understand the benefit that DNN-based noise reduction can provide to cochlear implant users. Although testing was completed at +5 dB signal to noise ratio for most patients, a ceiling effect was still present in the sentence recognition score measured for some of the bimodal cochlear implant patients. Testing at reduced signal-to-noise ratios is needed to further eliminate the ceiling effect and tease out differences between test conditions. While our study suggests that cochlear implant users significantly benefit from this technology in their acoustic ear, it is unknown how patients may benefit once this technology is available in their cochlear implant as well.

## 5. Conclusions

This study reveals that Deep Neural Network (DNN)-based noise reduction, when integrated into a hearing aid worn in the non-implanted ear, significantly improves speech understanding for bimodal cochlear implant users, especially in noisy environments with multiple talkers. The adoption of DNN technology in hearing aids offers a substantial increase in “bimodal benefit” compared to traditional hearing aids with standard signal processing. While the precise gain from DNN over conventional hearing aids may vary depending on the signal-to-noise ratio and the residual hearing in the non-implanted ear, the average bimodal benefit in noise with DNN technology was 40%, a significant improvement over the 21% observed with traditional hearing aid technology. Equipping the non-implanted ear with a DNN-based noise reduction hearing aid has the potential to alleviate the common complaint among cochlear implant users regarding difficulty understanding speech in background noise. Therefore, there is a clear need to incorporate DNN technology into hearing aids that are compatible with various cochlear implant manufacturers. Furthermore, integrating this technology into both the cochlear implant speech processor and a contralateral DNN-enabled hearing aid is likely to yield the best possible outcomes for bimodal cochlear implant patients.

## Figures and Tables

**Table 1:** Detailed demographics for each patient and for the total group. CI = cochlear implant; LF-PTA = low-frequency pure tone average.

Subject	Age (Years)	Duration of CI Use (Days)	LF-PTA	Sex
B1	86	43	32.5	M
B2	84	4523	56.25	M
B3	78	490	42.5	M
B4	89	40	63.75	M
B5	78	96	47.5	M
B6	83	1254	46.25	M
B7	83	833	55	M
B8	82	2714	52.5	F
B9	71	115	50	M
B10	73	366	41.25	M
B11	83	253	52.5	M
Mean	80	975	49	

## Data Availability

The original contributions presented in this study are included in the article. Further inquiries can be directed to the corresponding author.

## References

[1] Berg K.A., Noble J.H., Dawant B.M., Dwyer R.T., Labadie R.F., Gifford R.H. (2019). Speech recognition as a function of the number of channels in perimodiolar electrode recipients. J. Acoust. Soc. Am..

[2] Reynolds S.M., Gifford R.H. (2019). Effect of signal processing strategy and stimulation type on speech and auditory perception in adult cochlear implant users. Int. J. Audiol..

[3] Falcón González J.C., Borkoski Barreiro S., Ramos De Miguel A., Ramos Macías A. (2019). Improvement of speech perception in noise and quiet using a customised Frequency-Allocation Programming (FAP) method. Acta Otorhinolaryngol. Ital..

[4] Creff G., Lambert C., Coudert P., Pean V., Laurent S., Godey B. (2024). Comparison of Tonotopic and Default Frequency Fitting for Speech Understanding in Noise in New Cochlear Implantees: A Prospective, Randomized, Double-Blind, Cross-Over Study. Ear Hear..

[5] Holder J.T., Reynolds S.M., Sunderhaus L.W., Gifford R.H. (2018). Current Profile of Adults Presenting for Preoperative Cochlear Implant Evaluation. Trends Hear..

[6] Dunn C.C., Tyler R.S., Witt S.A. (2005). Benefit of wearing a hearing aid on the unimplanted ear in adult users of a cochlear implant. J. Speech Lang. Hear. Res..

[7] Gifford R.H., Dorman M.F., Sheffield S.W., Teece K., Olund A.P. (2014). Availability of binaural cues for bilateral implant recipients and bimodal listeners with and without preserved hearing in the implanted ear. Audiol. Neurootol..

[8] van Hoesel R.J.M. (2012). Contrasting benefits from contralateral implants and hearing aids in cochlear implant users. Hear. Res..

[9] Zhang T., Dorman M.F., Spahr A.J. (2010). Information from the voice fundamental frequency (F0) region accounts for the majority of the benefit when acoustic stimulation is added to electric stimulation. Ear Hear..

[10] Hoppe U., Hocke T., Digeser F. (2018). Bimodal benefit for cochlear implant listeners with different grades of hearing loss in the opposite ear. Acta Otolaryngol..

[11] Gifford R.H., Dorman M.F. (2019). Bimodal Hearing or Bilateral Cochlear Implants? Ask the Patient. Ear Hear..

[12] Neuman A.C., Waltzman S.B., Shapiro W.H., Neukam J.D., Zeman A.M., Svirsky M.A. (2017). Self-Reported Usage, Functional Benefit, and Audiologic Characteristics of Cochlear Implant Patients Who Use a Contralateral Hearing Aid. Trends Hear..

[13] Kessler D.M., Wolfe J., Blanchard M., Gifford R.H. (2020). Clinical Application of Spectral Modulation Detection: Speech Recognition Benefit for Combining a Cochlear Implant and Contralateral Hearing Aid. J. Speech Lang. Hear. Res..

[14] Illg A., Bojanowicz M., Lesinski-Schiedat A., Lenarz T., Büchner A. (2014). Evaluation of the bimodal benefit in a large cohort of cochlear implant subjects using a contralateral hearing aid. Otol. Neurotol..

[15] Sheffield S.W., Gifford R.H. (2014). The benefits of bimodal hearing: Effect of frequency region and acoustic bandwidth. Audiol. Neurootol..

[16] Firszt J.B., Reeder R.M., Holden L.K., Dwyer N.Y., Asymmetric Hearing Study Team (2018). Results in Adult Cochlear Implant Recipients With Varied Asymmetric Hearing: A Prospective Longitudinal Study of Speech Recognition, Localization, and Participant Report. Ear Hear..

[17] Powers T., Beilin J. (2013). True advances in hearing aid technology: What are they and where’s the proof. Hear. Rev..

[18] Mueller H.G., Weber J., Bellanova M. (2011). Clinical evaluation of a new hearing aid anti-cardioid directivity pattern. Int. J. Audiol..

[19] Froehlich M., Freels K., Powers T.A. (2015). Speech recognition benefit obtained from binaural beamforming hearing aids: Comparison to omnidirectional and individuals with normal hearing. Audiol. Online.

[20] McCreery R.W., Venediktov R.A., Coleman J.J., Leech H.M. (2012). An evidence-based systematic review of directional microphones and digital noise reduction hearing aids in school-age children with hearing loss. Am. J. Audiol..

[21] Michels A., Oukheira Y., Brendel M., Aschendorff A., Arndt S., Wesarg T. (2022). Effect of adaptive beamforming and noise reduction algorithms on speech intelligibility and noise tolerance in bimodal cochlear implant users. Cochlear Implant. Int..

[22] Stronks H.C., Briaire J., Frijns J. (2022). Beamforming and Single-Microphone Noise Reduction: Effects on Signal-to-Noise Ratio and Speech Recognition of Bimodal Cochlear Implant Users. Trends Hear..

[23] Alcántara J.L., Moore B.C., Kühnel V., Launer S. (2003). Evaluation of the noise reduction system in a commercial digital hearing aid. Int. J. Audiol..

[24] Brons I., Houben R., Dreschler W.A. (2015). Acoustical and Perceptual Comparison of Noise Reduction and Compression in Hearing Aids. J. Speech Lang. Hear. Res..

[25] Raufer S., Kohlhauer P., Uhlemayr F., Kühnel V. Deep learning-based denoising for hearing aid applications. Proceedings of the International Hearing Aid Conference (IHCON).

[26] Saoji A.A., Sheikh B.A., Bertsch N.J., Goulson K.R., Graham M.K., McDonald E.A., Bross A.E., Vaisberg J.M., Kühnel V., Voss S.C. (2024). How Does Deep Neural Network-Based Noise Reduction in Hearing Aids Impact Cochlear Implant Candidacy?. Audiol. Res..

[27] Hasemann H., Krylova A. (2024). Spheric Speech Clarity: Enhancing Understanding in Everyday Listening Situations. Phonak. https://www.phonak.com/content/dam/phonak/en/evidence-library/white-paper/technical-paper/PH_Insight_SphericSpeechClarity_210x297_EN_028-2684-02_V1.00.pdf.

[28] Kong Y.-Y., Carlyon R.P. (2007). Improved speech recognition in noise in simulated binaurally combined acoustic and electric stimulation. J. Acoust. Soc. Am..

[29] Brown C.A., Bacon S.P. (2009). Low-frequency speech cues and simulated electric-acoustic hearing. J. Acoust. Soc. Am..

[30] Li N., Loizou P.C. (2008). A glimpsing account for the benefit of simulated combined acoustic and electric hearing. J. Acoust. Soc. Am..

[31] Berrettini S., Passetti S., Giannarelli M., Forli F. (2010). Benefit from bimodal hearing in a group of prelingually deafened adult cochlear implant users. Am. J. Otolaryngol..

